# Supplementary material for: Targeted Delivery of Antifungal Liposomes to Rhizopus delemar
Source: J Fungi (Basel). 2022 Mar 30;8(4):352. doi: 10.3390/jof8040352 (PMC9026866; doi:10.3390/jof8040352)

Supplemental Figures

Supplementary Figure S1. Preparing samples of *R. delemar* growing on the surface of agar plugs for top down epifluorescence microscopy. Sporangiospores were germinated and grown to different developmental stages on the surface of agar plates in RPMI-MOPS media. Seven-mm circular plugs were removed with a sterile cork-borer, transferred to 24-well microtiter plates, and washed once submerged in PBS. The cells were fixed in 3.7% formaldehyde in PBS for 1 hr or left live, and washed thrice in PBS. The details of the liposome staining protocol is given in the text.

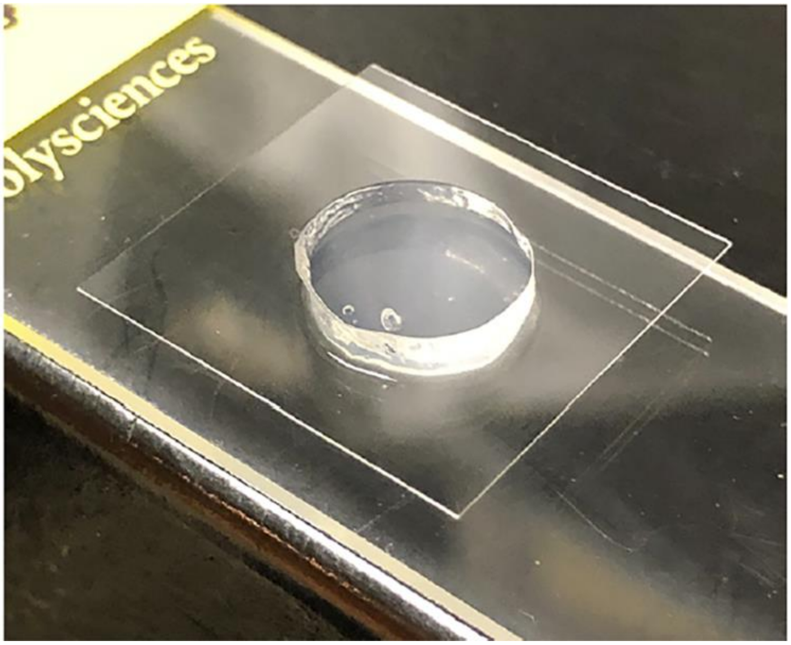

Supplementary Figure S2. The quantification of DEC1-AmB-LL binding to fixed and live cells relative to control liposome binding. Panels **A** and **B** in this figure show biological replicates of the experiments in **Figure 2D** and **2F.** See the legend to **Figure 2** for details. Panel **C** shows the labeling of *R. delemar* hyphae with rhodamine B-conjugated Dectin-1, DEC1-Rhod, photographed at 5x and 63x using epifluorescence. CW-stained hyphae are shown in green, and DEC1-Rhod staining is shown in red. Size bars indicate 400 and 20 micron scales for the 5x and 63x images, respectively.

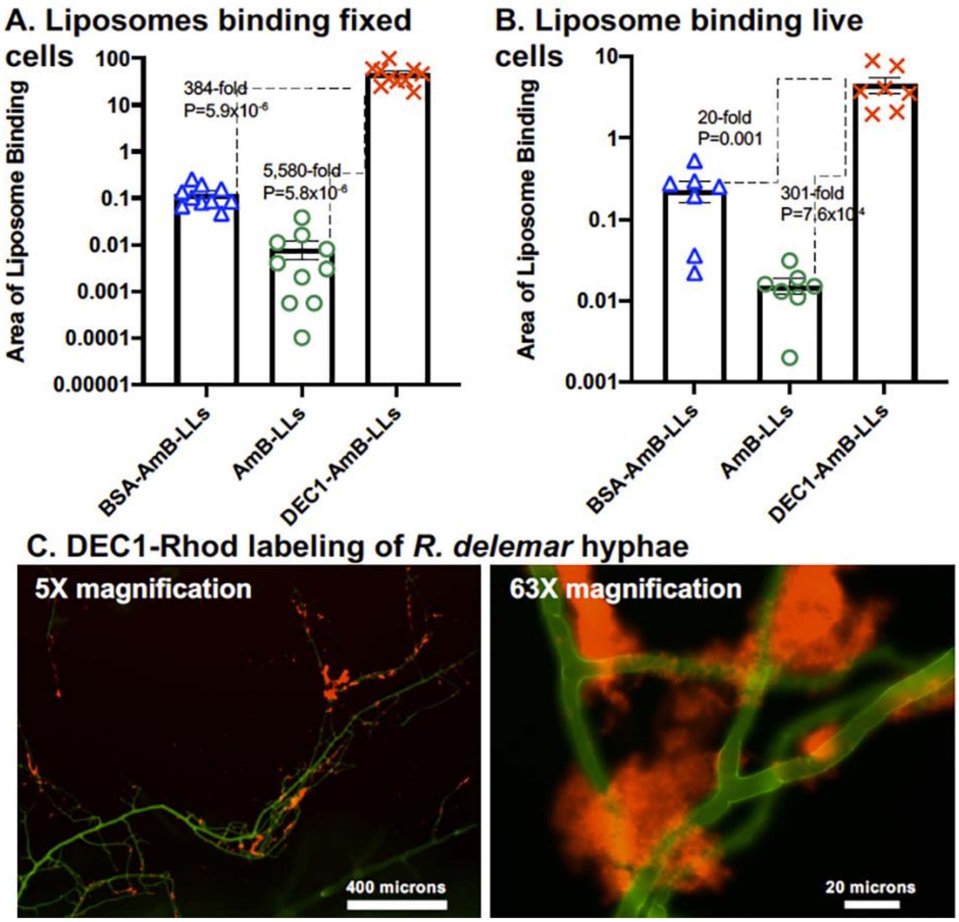

Supplementary Figure S3. The beta-glucan specificity of Dectin-1-targeted liposomes, DEC1-AmB-LLs, binding to *R. delemar.* This figure shows a biological replicate of the experiment in **Figure 3D.** See the legend to **Figure 3** for details.

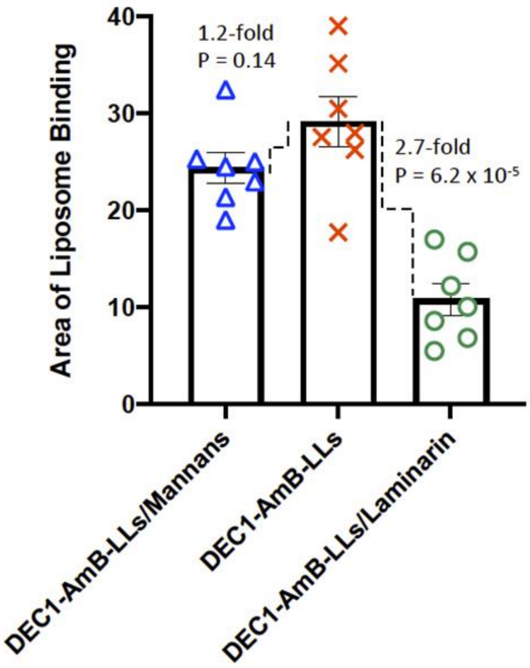

Supplementary Figure S4. Inhibition and killing assays based on cell growth and density. Panels **A** and **B** in this figure show biological replicates of the experiments in **Figure 4A** and **4B**, respectively. See the legend to **Figure 4** for details.

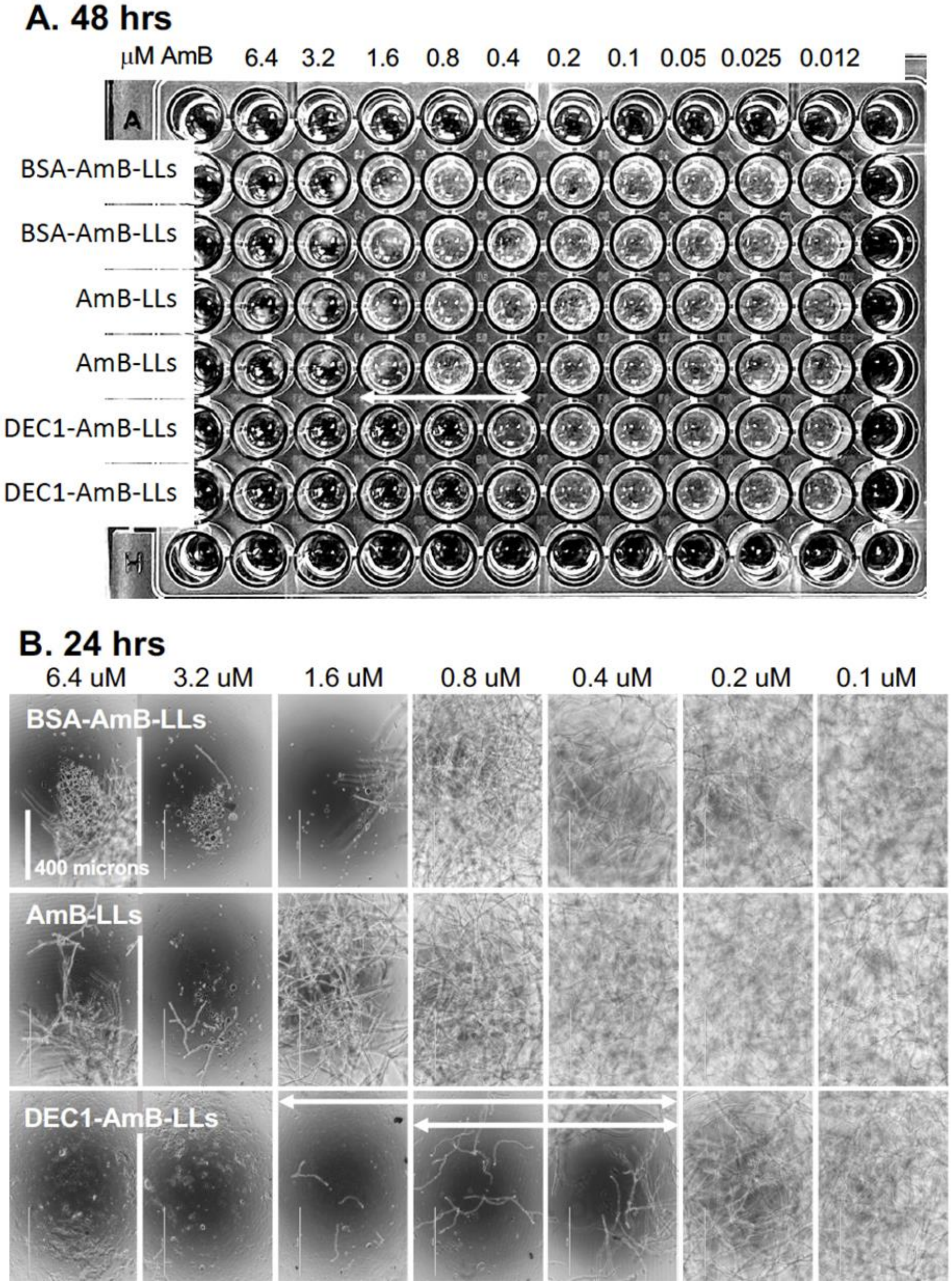

Supplementary Figure S5. Inhibition and killing assays based on cell density and metabolic activity. Panel **A** shows the residual metabolic activity of cells treated with targeted and untargeted liposomes delivering a wide range of AmB concentrations for 24 hrs. Panels **B** and **C** show replicates of the cell density and CTB assay experiments in **Figure 5A** and **5B.** Panel **D** shows a replicate of the CTB assay in **Figure 5C**. See the legends in the main text for details.

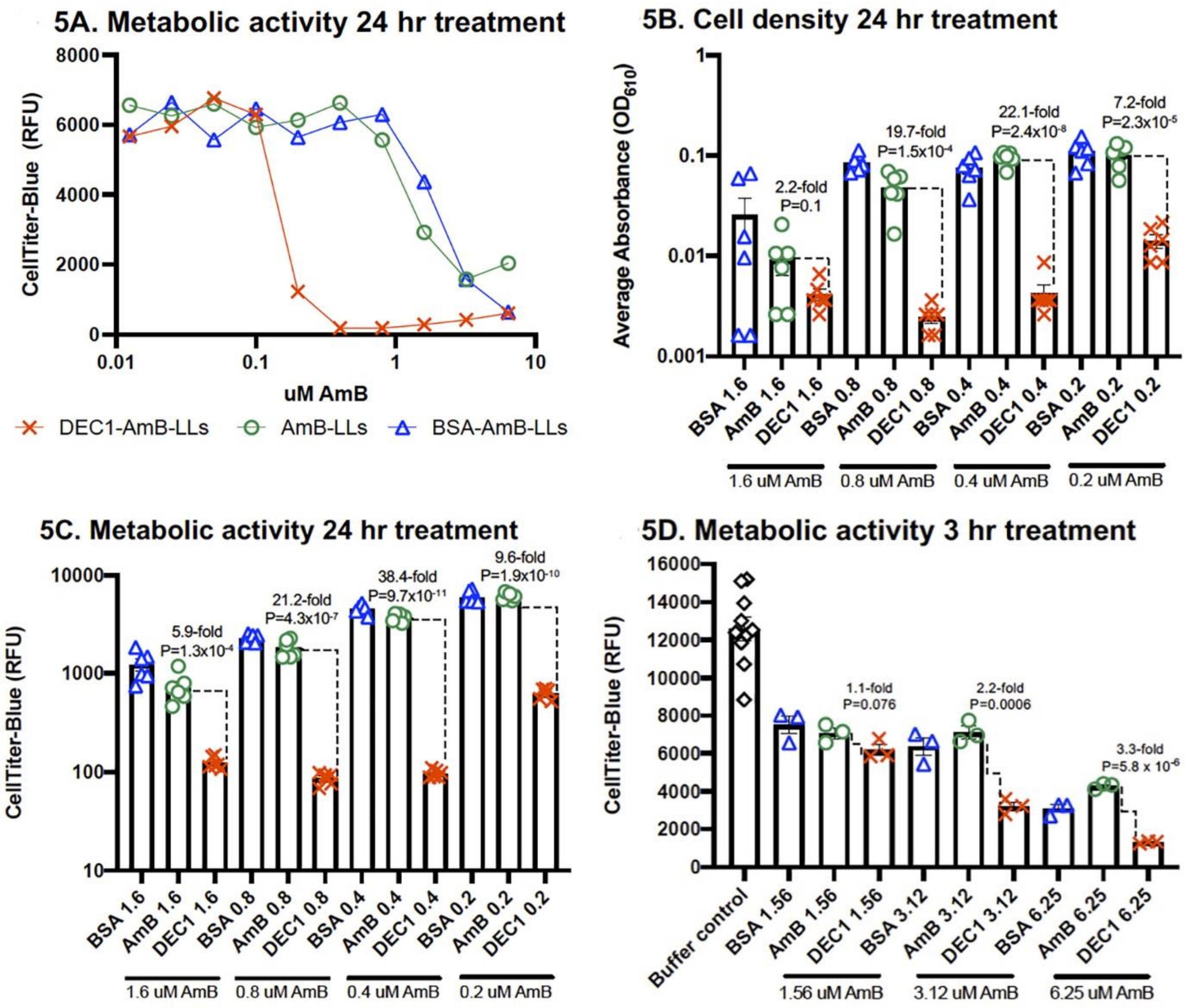

Supplement: Supplementary file 1 [file jof-08-00352-s001.zip › jof-1644546-supplementary.pdf]
